# Doctoral nursing education in east and Southeast Asia: characteristics of the programs and students’ experiences of and satisfaction with their studies

**DOI:** 10.1186/s12909-020-02060-1

**Published:** 2020-05-08

**Authors:** Alex Molassiotis, Tao Wang, Huong Thi Xuan Hoang, Jing-Yu Tan, Noriko Yamamoto-Mitani, Karis F. Cheng, Josefina A. Tuazon, Wipada Kunaviktikul, Lorna K. P. Suen

**Affiliations:** 1 School of Nursing, The Hong Kong Polytechnic University, Kowloon, Hong Kong SAR; 2 Faculty of Nursing, Phenikaa University, Hanoi, Vietnam; 3 College of Nursing and Midwifery, Charles Darwin University, Darwin, Australia; 4 Department of Health Sciences & Nursing, The University of Tokyo, Tokyo, Japan; 5 Alice Lee Centre for Nursing Studies, Yong Loo Lin School of Medicine, National University of Singapore, Singapore, Singapore; 6 College of Nursing, University of the Philippines Manila, Manila, Philippines; 7 Faculty of Nursing, Chiang Mai University, Chiang Mai, Thailand

**Keywords:** Doctoral education, PhD degree, Nursing, Asia, Student experience

## Abstract

**Background:**

The characteristics of nursing doctoral programs and the doctoral students’ experience have not been thoroughly investigated. Hence, this study aimed to describe the characteristics of nursing doctoral programs in East and South East Asian (ESEA) countries and regions from the views of doctoral program coordinators, and to explore the students’ experiences of and satisfaction with their doctoral nursing program.

**Methods:**

A cross-sectional survey was conducted using two self-designed questionnaires, one focusing on PhD program coordinators and the other on doctoral students. Characteristics of the nursing doctoral programs focused on *program characteristic*s, *faculty characteristics*, *career pathways for graduates*, and *challenges for nursing doctoral education*. Doctoral students’ assessment of study experiences included *quality of supervision, doctoral training programs*, *intellectual/cultural climate of institutions*, *general facilities/support*, and *the overall study experience and satisfaction*.

**Results:**

In the PhD coordinators survey, 46 institutions across nine ESEA countries and regions participated. More than half of nursing departments had academic members from other health science disciplines to supervise doctoral nursing students. The majority of graduates were holding academic or research positions in higher education institutions. Faculty shortages, delays in the completion of the program and inadequate financial support were commonly reported challenges for doctoral nursing education. In the students’ survey, 193 doctoral students participated. 88.3% of the students were satisfied with the supervision they received from their supervisors; however, 79% reported that their supervisors ‘pushed’ them to publish research papers. For doctoral training programs, 75.5% were satisfied with their curriculum; but around half reported that the teaching training components (55.9%) and mobility opportunities (54.2%) were not included in their programs. For overall satisfaction with the intellectual and cultural climate, the percentages were 76.1 and 68.1%, respectively. Only 66.7% of the students felt satisfied with the facilities provided by their universities and nursing institutions.

**Conclusion:**

Doctoral nursing programs in most of the ESEA countries value the importance of both research and coursework. Doctoral nursing students generally hold positive experiences of their study. However, incorporating more teaching training components, providing more opportunities for international mobility, and making more effort to improve research-related facilities may further enhance the student experience. There is also a need to have international guidelines and standards for quality indicators of doctoral programs to maintain quality and find solutions to global challenges in nursing doctoral education.

## Background

An increasing trend in doctoral nursing education has occurred internationally in response to advances in healthcare technology and multidisciplinary initiatives to enhance the quality of care [1].. According to the Institute of Medicine and the Robert Wood Johnson Foundation [2], the number of doctoral-prepared nurses in the United States should be doubled by the year 2020 to meet the rapidly increasing needs for high quality healthcare, education and research. A significant growth of the doctoral nursing programs in United States has been seen over the past decade, with the number of institutions offering research-oriented and practice-focused doctoral nursing programs reaching 133 and 241 in 2013, respectively [3]. In Asia, expansion of doctoral nursing education has also been noted; our own search through personal contacts in each country, country e-lists available and website searches, revealed 196 universities from 11 South and South East Asian countries providing doctoral nursing education in 2017 (Table 1). From a global perspective, the accurate number of institutions offering doctoral nursing programs has not been reported, but we can infer that by now it must far exceed the number of 333, which was the latest data reported by The International Network of Doctoral Education in Nursing (INDEN) in 2013 [4].

**Table 1:** **Doctoral Nursing Programmes in South and South East Asia Countries** (as of May 2017)

Country or Region	No. of Universities with Doctoral Nursing Education	Type of Programme	
		Ph.D. Programme	Professional Doctorate Programme
**Hong Kong**	3	3	3
**Mainland China**	34	34	0
**Taiwan**	11	11	0
**Singapore**	1	1	0
**Japan**	84	84	0
**South Korea**	32	32	0
**Thailand**	7	7	1
**Malaysia**	8	7	1
**Philippines**	14	11	3
**Indonesia**	1	1	0
**Brunei**	1	1	0
**Total**	**196**	**192**	**8**

The rapid growth of doctoral nursing education brings up another issue regarding the quality of that education. Evaluation of the doctoral nursing programs is a highly-recommended approach to maintaining the quality of such programs [5, 6]. Literature about this issue is limited in Asia. Only a few publications described challenges of doctorate programs in specific Asian countries such as Japan, Korea, Taiwan, and Thailand. Challenges for nursing research-based doctoral education included shortages of qualified faculty, aging faculty, and concerns about the quality of the doctoral training [7]. Views and experiences from current and former students, academic staff members and the employers are crucial for a comprehensive evaluation of doctoral nursing education [5]. Experiences of and satisfaction with the education programs from the students’ perspectives have been regarded as an important indicator for high-quality postgraduate programs [8], and many relevant studies have been performed in universities outside Asian countries, such as universities in Australia [8–10] and in the United Kingdom [11]. According to van den Schoot [12], Harman [10] and the 2007 report from Macquarie University [8], many factors associated with the performance and quality of postgraduate education were identified, including sufficient information support before and at the commencement of the postgraduate study, sufficient facilities and work stations for supporting research studies, adequate administrative support, available financial support for research activities, etc. However, no study has been conducted so far to evaluate doctoral nursing programs in Asian countries. Therefore, the aim of the current study was to explore the characteristics of nursing doctoral education programs and the students’ experiences of and satisfaction with those programs in East and South East Asian (ESEA) countries and regions.

## Methods

### Study design

This study utilized a cross-sectional design using either a paper or open online questionnaire for data collection through a convenience sampling.

### Participants and procedures

The universities that offer doctoral nursing education (including both Doctor of Philosophy [PhD] programs and professional doctorate programs) across ESEA countries or regions (most being members of the East Asian Forum of Nursing Scholars [EAFONS], http://eafons.org/) were invited to participate in the survey. This survey included two parts: part one was an online survey regarding the characteristics of the nursing doctoral program and part two was an online and paper-based survey on students’ self-reported experience of and satisfaction with these programs.

For part one, an invitation email attached with a questionnaire was sent to the dean/head/director of nursing schools/departments in each university. The dean/head/director of nursing was invited to forward the questionnaire to the doctoral program coordinator(s) for completion. For part two, nursing doctoral students who attended the 20th East Asian Forum of Nursing Scholars (EAFONS) conference held in March 2017 were invited to participate in a survey; completed paper questionnaires were collected at the end of the conference. An identical online questionnaire in English language together with a survey invitation letter were later emailed to the dean/head/director of nursing in each university for distribution among their doctoral students. This was done to further enlarge the survey sample and enhance the representativeness of the survey findings, given that only a relatively small number of doctoral students attended the EAFONS conference. This was done from May 2017 to December 2017. Students who completed the questionnaire during the conference were asked not to complete the online form. Two follow-up emails were sent to increase the response rate. Ethical approval for this survey was obtained from the Human Subjects Ethics Sub-committee of the Hong Kong Polytechnic University. Participants were informed of the purpose of the study, who the investigators were and how data would be analysed and presented, assuring them of anonymity. Completion of the survey questionnaire implied consent.

The usability and technical functionality of the electronic questionnaire was tested with five students whose data was not included in the main study. Consistency and completeness checks were carried out before the questionnaire was submitted online. Duplicate entries were avoided by having a unique ID number for each participant who could not complete the survey more than once, controlled through their IP address. Participants had also the option, through a ‘back’ button, to review their responses and change them before final submission to the system.

### Survey questionnaires

#### A. Questionnaire for the coordinators of the doctoral training programs

This questionnaire was designed to collect data regarding the doctoral program features and relevant faculty information for doctoral supervision based on past literature [13, 14] and expert panel discussions with senior academic members of EAFONS. This questionnaire comprised of 5 pages with 56 items and five modules including characteristics of institutions (5 questions), program characteristics (28 questions), faculty characteristics (12 questions), career pathways for nursing doctoral graduates (9 questions), and challenges for nursing research doctorate programs (3 questions). The latter three questions were open-ended and were used to explore the challenges faced in nursing research doctoral programs, with questions being “What are the main challenges that your institution may face in terms of the program features?”; “What are the main challenges that your institution may face in terms of the quality of faculty for the supervision of nursing research doctoral students?”, “In addition to the challenges mentioned above, does your institution face any other challenges regarding the nursing research doctoral program?”

#### B. Questionnaire for doctoral students

This questionnaire was designed to assess doctoral students’ experiences of and satisfaction with their doctoral nursing programs. It was developed on the basis of previous literature/questionnaires [8, 14–19] and expert panel discussions with senior academic members of EAFONS. The questionnaire consisted of 7 pages with 64 items and 6 modules, including general information about the doctoral students (9 items), quality of supervision (17 items), doctoral training program (9 items), intellectual/cultural climate (13 items), general facilities support (12 items), and overall study experiences and satisfaction (4 items). Among these 64 items, 60 were closed-ended questions and four were open-ended questions. For the closed questions, a six-point Likert-type scale (“very much agree”, “agree”, “neutral”, “disagree”, “very much disagree” and “no experience”) was used to rate and measure the extent to which doctoral students agreed or disagreed on the content mentioned in each item. For the open-ended questions, one question was about the curriculum of their doctoral training program, (“what are the compulsory and elective subjects in your doctoral program?”), while the other three were about evaluating their overall study experiences, and the questions were “what is your biggest gain in the nursing doctoral study at your current institution?”, “what is the biggest challenge in the nursing doctoral study at your current institution?”, and “if your institution could make some improvements in the facilities offered to you, what improvements you would like to see?”. The reliability coefficients (Cronbach alpha) of the different sections ranged from 0.89 (section on satisfaction with doctoral training) to 0.95 (section on satisfaction with supervision), while the overall Cronbach alpha of the sections was high at 0.947.

### Data analysis

For closed questions, descriptive analysis was adopted by calculating the percentages of respondents. For open-ended questions, simple content analysis was used to describe and quantify the data, developing categories based on the frequency of extracted codes [20].From some universities we received submitted surveys from several coordinators (part I, PhD program coordinators survey). Hence the quantitative data for this part were analyzed from one coordinator in each institution only, as all the questionnaires from the same site contained identical information. Finally, responses from 20 institutions were fully analyzed in the coordinators survey. Responses from the other 26 institutions with overlapping answers were removed from the quantitative analysis but the open ended questions, reflecting personal experiences and ideas, were included in the qualitative analysis. For the data of part two (students experience survey), both full and partial responses were used in the analysis. The options of “very much agree” and “agree” were recorded as a single unit, and the same for “disagree” and “very much disagree”.

## Results

### Demographic characteristics of the respondents

Seventy-six coordinators from 46 institutions offering doctoral nursing programs in nine East and South East Asian countries/regions participated in part one survey (coordinators survey). There were 20 fully completed surveys and 56 partially completed. Japan had the largest number of institutions participating (30.4%) followed by China (17.4%), Thailand, South Korea, Taiwan, Malaysia, the Philippines, Hong Kong, and Singapore (Table 2). The majority of coordinators were current supervisors for nursing research doctoral students (81.6%). Furthermore, 193 doctoral students from 41 of the 46 institutions participated in part two survey (students’ survey) (Table 2). In this part, there were 135 full responses and 58 responses that omitted to complete specific sections of the questionnaire. Approximately two-fifths of the students were from universities in Japan and around one-fifth from universities in Thailand. For the home countries of the respondents, nearly all of the doctoral students came from Asian countries, along with very few students coming from Africa (1.6%). The majority of the students were female (83.9%) and full-time students (66.8%). The majority were PhD students while 8% were studying for a professional doctorate program (Doctor of Nursing or Doctor of Health Sciences).

**Table 2:** Participants’ country of origin

Country	Coordinators (N, %)	Doctoral students (N, %)
Japan	40 (52.7)	76 (39.3)
China	8 (10.5)	16 (8.3)
Taiwan	8 (10.5)	10 (5.2)
Philippines	6 (7.9)	9 (4.7)
Thailand	5 (6.6)	38 (19.7)
Malaysia	4 (5.3)	2 (1.0)
South Korea	3 (3.9)	18 (9.3)
Singapore	1 (1.3)	3 (1.6)
Hong Kong	1 (1.3)	18 (9.3)
No answer	0	3 (1.6)

### Characteristics of the nursing doctoral education programs

#### Program characteristics (*n* = 20 institutions)

There were 510 current nursing doctoral students enrolled among these institutions. The average annual intake of nursing doctoral students in each institution was 5.7 ± 4.3 students (min = 1; max = 15). In some institutions students from outside the nursing profession could be enrolled (although all our sample was with students who were qualified nurses). Most institutions required a Master’s degree before enrolling into a PhD program. The normal study period of the programs was three to 5 years for full-time students and three to more than 6 years for part-time students. Less than half of the full-time (46.5%) and part-time (39.8%) students graduated within the normal study period. Full financial support during the normal study period was provided in half of the institutions, and 75% of the institutions provided additional financial support to full-time students for data collection and attending local/overseas conferences.

Coursework was required in 85% of the participating institutions. Regarding the study areas of the programs, 37 areas were listed and the commonly reported areas were mental health and psychiatric nursing, gerontology nursing and community health nursing. Research methodology-related subjects were the most common compulsory subjects as well as elective subjects. Other common compulsory subjects were nursing science/philosophy, theory development in nursing and health promotion-related courses (Table 3). English language was used in 30% of the institutions, and 10% of them used dual language (English and native language). Publication requirements for the award of doctorate degree were reported by 75% of the participating institutions, with 60% of them requiring publication in an international peer-reviewed journal. Regarding collaborative training, a quarter of the responding institutions had domestic collaborations and 60% had international collaborations. Those collaborations included student exchanges, joint doctorate programs (or joint supervision with other institutions) and internship programs.

**Table 3:** Compulsory and elective subjects reported in the nursing research doctorate programs

Compulsory subjects	Number of institutions including this subject in their program
Research Methodology Courses	20
Nursing science/Philosophy	7
Theory development/Construction in nursing	6
Research seminar	4
Health system and policy courses	4
Research Ethics	3
English course for Doctorate students	3
Marxist Philosophy	2
Maternal Nursing	2
Practicum	1
Guided-study	1
Islamic Input for Health Profession	1
Community Health Nursing	1
**Elective subjects**	
Research Methodology Courses	9
Instrument Development for Nursing Research	5
Courses related to Health Promotion	4
English courses	3
Courses related to chronic illness	3
Courses related to Family Health	3
Nursing Therapeutics	3
Teaching skill courses	2
Continuity care	2
Information technology	2
Clinical Humanity & Nursing Healing	2
Guided Study	1
Advanced Nursing Practice	1
Traditional Chinese Medicine theories and practice	1
Nursing science	1
Nursing science	1
Advance Pediatric nursing Science	1
Advance studies in International Communication	1
Theoretical Nursing (Advance)	1
Philosophical Anthropology	1
Nursing Pathophysiology	1
Biobehavioral Nursing	1
Advanced International Community Health Nursing	1
Disaster Nursing Theory & Practice	1
Advanced Course in Global Health and Nursing	1

#### Faculty characteristics (*n* = 20 institutions)

The total number of faculty members in the 20 nursing institutions participating in the survey was 922 staff members, and the average number of faculty members in each institution was 46.1 ± 30; 51% of them held a research doctorate degree, among which, 39.2% had received their doctorate degree from overseas, whereas the remaining had a Master’s degree only or less. More than half of the institutions had academic members with non-nursing health science background supervising doctoral nursing students (56%), in most cases with no nursing faculty as supervisors; the most common disciplines were medical sciences-related; pharmacology and psychology. 302 full-time faculty members (about one-third of the total number of faculty members) were qualified for supervision of nursing research doctoral students. The eligibility criteria for a full-time faculty member to be a supervisor were academic qualifications (44.1%), publications (14.7%), academic rank or achievements (32.4%), teaching or supervision experience (5.9%) and being principal investigator of a project or research grant (2.9%). In nearly half of the institutions, the maximum number of doctoral nursing students assigned for each chief-supervisor was 1 to 5 students (45%), one third of faculty had the maximum doctoral student per chief-supervisor as 6–10 students and a quarter of them had no limit for the maximum supervision load.

#### Career pathway for nursing research doctoral graduates (*n* = 20)

The participating institutions reported 738 PhD graduates from the start of their program up to the time they completed the survey. Among them, 447 nursing research doctoral graduates (60%) were holding academic positions with 9.4% of them working overseas. 110 graduates were currently holding research positions with 37.3% of them working at overseas institutions. 81 graduates were currently holding clinical positions and 24.7% of them were working at overseas hospitals.

#### Challenges for nursing research doctoral education (*n* = 76)

The majority of the coordinators reported challenges regarding program features (81.1%). The most commonly mentioned challenge was the “size and length of the program” (20.5%), followed by “collaborative opportunities for training research doctoral students” (15.4%), “research and coursework components in the program” (12.8%), and “sufficient supply of supervisors, facilities and support” (17.4%). Other challenges were “quantity and quality of faculty members,” “financial support,” “publication requirements,” “competition,” and “future development” of the programs (23.1%).

Regarding issues affecting the faculty members’ perception of the quality of their student supervision, the most common response was “supervision workload” (33.3%), followed by “lack of supervision quality assurance for research doctorate programs” (29.2%), “educational background of the faculty members” (12.5%), “lack of adequate PhD supervisors” (16.7%) and “lack of time for research” (8.3%).

### Students’ experiences of and satisfaction with their study

#### Supervision quality

Response rates for each item within this domain were above 83.5%. Most of the students (88.3%) were generally satisfied with the supervision quality. Detailed information is presented in Table 4.

**Table 4:** Student experiences and satisfaction with aspects of their PhD studies

Supervision quality						
Item	Content of Item	No. of respondents (N, %)	Very much agree + Agree (N, %)	Neutral (N, %)	Disagree + Very much disagree (N, %)	No experience (N, %)
**II-1**	The frequency of the supervision meetings with my supervisor(s) is reasonable.	163/194 (84.0%)	135/163 (82.8%)	21/163 (12.9%)	7/163 (4.3%)	0 (0%)
**II-2**	My supervisor(s) can maintain the supervision meetings on a regular basis.	163/194 (84.0%)	127/163 (77.9%)	23/163 (14.1%)	13/163 (8.0%)	0 (0%)
**II-3**	My supervisor(s) can give me timely feedback when I need professional support.	163/194 (84.0%)	138/163 (84.7%)	19/163 (11.7%)	6/163 (3.6%)	0 (0%)
**II-4**	The quality of the supervision meetings with my supervisor (s) is high.	162/194 (83.5%)	136/162 (84.0%)	20/162 (12.3%)	6/162 (3.7%)	0 (0%)
**II-5**	My supervisor(s) have sufficient expertise in my doctoral research topic.	162/194 (83.5%)	126/162 (77.8%)	25/162 (15.4%)	8/162 (4.9%)	3/162 (1.9%)
**II-6**	My supervisor(s) can give me appropriate guidance for my doctoral research topic selection.	162/194 (83.5%)	137/162 (84.6%)	16/162 (9.8%)	6/162 (3.7%)	3/162 (1.9%)
**II-7**	My supervisor(s) have sufficient expertise in the methodology that I utilized in my doctoral project.	162/194 (83.5%)	138/162 (85.2%)	11/162 (6.8%)	9/162 (5.6%)	4/162 (2.4%)
**II-8**	My supervisor(s) can give me useful feedback on my doctoral study progress.	162/194 (83.5%)	140/162 (86.4%)	17/162 (10.5%)	3/162 (1.9%)	2/162 (1.2%)
**II-9**	My supervisor(s) have the awareness of developing my research capacities such as critical thinking.	162/194 (83.5%)	150/162 (92.6%)	8/162 (4.9%)	4/162 (2.5%)	0 (0%)
**II-10**	My supervisor(s) can give me very clear direction and guidance for my doctoral research project.	162/194 (83.5%)	135/162 (83.3%)	21/16 (13.0%)	6/162 (3.7%)	0 (0%)
**II-11**	My supervisor(s) act as a good academic role model and mentor.	162/194 (83.5%)	147/162 (90.8%)	8/162 (4.9%)	7/162 (4.3%)	0 (0%)
**II-12**	My supervisor(s) usually give me a lot of encouragement and help build my confidence.	162/194 (83.5%)	136/162 (84.0%)	20/162 (12.3%)	6/162 (3.7%)	0 (0%)
**II-13**	My supervisor(s) usually help me face challenges and difficulties related to my study.	162/194 (83.5%)	135/162 (83.3%)	22/162 (13.6%)	5/162 (3.1%)	0 (0%)
**II-14**	My supervisor(s) push me to publish research papers.	162/194 (83.5%)	128/162 (79.0%)	19/162 (11.7%)	9/162 (5.6%)	6/162 (3.7%)
**II-15**	My supervisor(s) are willing to give me advices on research issues.	162/194 (83.5%)	138/162 (85.2%)	17/162 (10.5%)	5/162 (3.1%)	2/162 (1.2%)
**II-16**	My supervisor(s) are willing to give me advice on my career development.	162/194 (83.5%)	127/162 (78.4%)	26/162 (16.1%)	8/162 (4.9%)	1/162 (0.6%)
**II-17**	**Overall,** I am satisfied with the quality and effectiveness of my doctoral supervision.	162/194 (83.5%)	143/162 (88.3%)	11/162 (6.8%)	7/162 (4.3%)	1/162 (0.6%)
**Training program**						
**III-6**	Do you agree the doctoral training at your institution enhances your critical thinking skills?	140/194 (72.2%)	115/140 (82.1%)	21/140 (15.0%)	3/140 (2.2%)	1/140 (0.7%)
**III-7**	Do you feel confident about writing proposal for funding application after receiving the doctoral trainings?	141/194 (72.7%)	83/141 (58.9%)	37/141 (26.2%)	17/141 (12.1%)	4/141 (2.8%)
**III-8**	Do you feel confident about research project management after receiving the doctoral trainings?	141/194 (72.7%)	95/141 (67.4%)	25/141 (17.7%)	17/141 (12.1%)	4/141 (2.8%)
**III-9**	Overall, do you feel satisfied with the doctoral training at your institution?	141/194 (72.7%)	108/141 (76.6%)	24/141 (17.0%)	6/141 (4.3%)	3/141 (2.1%)
**Intellectual and cultural climate**						
**IV-1**	My institution provides opportunities for research interactions with peer doctoral students from other universities.	137/194 (70.6%)	85/137 (62.0%)	22/137 (16.1%)	21/137 (15.3%)	9/137 (6.6%)
**IV-2**	I feel I belong to the academic community in my institution.	138/194 (71.1%)	87/138 (63.0%)	29/138 (21.0%)	16/138 (11.6%)	6/138 (4.4%)
**IV-3**	Apart from my supervisor(s), I can get professional support from other academic staff members in my institution.	138/194 (71.1%)	104/138 (75.3%)	24/138 (17.4%)	7/138 (5.1%)	3/138 (2.2%)
**IV-4**	I can get support from other peer doctoral students in my institution.	138/194 (71.1%)	108/138 (78.2%)	19/138 (13.8%)	8/138 (5.8%)	3/138 (2.2%)
**IV-5**	The academic staff members and doctoral students in my institution are willing to share their research with peers.	138/194 (71.1%)	103/138 (74.6%)	23/138 (16.7%)	10/138 (7.2%)	2/138 (1.5%)
**IV-6**	My institution encourages academic interactions among peer doctoral students.	138/194 (71.1%)	104/138 (75.4%)	21/138 (15.2%)	10/138 (7.2%)	3/138 (2.2%)
**IV 7**	Regular research seminars/forums are encouraged and held in my institution.	138/194 (71.1%)	112/138 (81.2%)	17/138 (12.3%)	6/138 (4.3%)	3/138 (2.2%)
**IV 8**	The working environment in my institution is very supportive.	138/194 (71.1%)	102/138 (73.9%)	27/138 (19.6%)	6/138 (4.3%)	3/138 (2.2%)
**IV-9**	The academic staff and doctoral students in my institution are culturally diversified.	138/194 (71.1%)	95/138 (68.8%)	30/138 (21.7%)	11/138 (8.0%)	2/138 (1.5%)
**IV-10**	My institution is inclusive of people with different cultural backgrounds.	138/194 (71.1%)	96/138 (69.6%)	26/138 (18.8%)	13/138 (9.4%)	3/138 (2.2%)
**IV-11**	My institution encourages cultural interactions among peer doctoral students.	138/194 (71.1%)	94/138 (68.1%)	35/138 (25.4%)	6/138 (4.3%)	3/138 (2.2%)
**IV-12**	**Overall,** I am satisfied with the intellectual climate in my institution.	138/194 (71.1%)	105/138 (76.1%)	28/138 (20.3%)	4/138 (2.9%)	1/138 (0.7%)
**IV-13**	**Overall,** I am satisfied with the cultural climate in my institution.	138/194 (71.1%)	94/138 (68.1%)	32/138 (23.2%)	10/138 (7.2%)	2/138 (1.5%)
**Facilities**						
**V-1**	There are sufficient academic library resources (e.g. holdings, electronic databases and search tools) in my university.	138/194 (71.1%)	112/138 (81.2%)	16/138 (11.6%)	9/138 (6.5%)	1/138 (0.7%)
**V-2**	My university library staff can provide appropriate technical and professional supports to doctoral students.	138/194 (71.1%)	107/138 (77.5%)	18/138 (13.1%)	10/138 (7.2%)	3/138 (2.2%)
**V-3**	My university library can provide comfortable and convenient study environment to doctoral students.	138/194 (71.1%)	103/138 (74.6%)	19/138 (13.8%)	12/138 (8.7%)	4/138 (2.9%)
**V-4**	Library facilities in my university can well support my doctoral study.	138/194 (71.1%)	104/138 (75.4%)	26/138 (18.8%)	7/138 (5.1%)	1/138 (0.7%)
**V-5**	My institution provides the doctoral students with sufficient space for teaching and research activities.	138/194 (71.1%)	100/138 (72.5%)	28/138 (20.3%)	9/138 (6.5%)	1/138 (0.7%)
**V-6**	My institution has sufficient administrative and technical staff to support student activities.	138/194 (71.1%)	88/138 (63.8%)	34/138 (24.6%)	13/138 (9.4%)	3/138 (2.2%)
**V-7**	My institution can provide necessary equipment and resources for doctoral research activities.	138/194 (71.1%)	95/138 (68.8%)	31/138 (22.5%)	11/138 (8.0%)	1/138 (0.7%)
**V-8**	The admission and enrolment procedures for doctoral students in my institution are clear and easy to access.	138/194 (71.1%)	102/138 (73.9%)	26/138 (18.8%)	9/138 (6.5%)	1/138 (0.7%)
**V-9**	Working stations (e.g. research student office) for doctoral students are well provided.	137/194 (70.6%)	80/137 (58.4%)	32/137 (23.4%)	20/137 (14.6%)	5/137 (3.6%)
**V-10**	Office supplies (e.g. computer, telephone and printer) are well provided.	138/194 (71.1%)	73/138 (52.9%)	36/138 (26.1%)	28/138 (20.3%)	1/138 (0.7%)
**V-11**	My institution has provided doctoral students with adequate financial support for research-related work.	138/194 (71.1%)	71/138 (51.4%)	35/138 (25.4%)	29/138 (21.0%)	3/138 (2.2%)
**V-12**	**Overall,** I am satisfied with the quality of facilities in my university and institution.	138/194 (71.1%)	92/138 (66.7%)	33/138 (23.9%)	13/138 (9.4%)	0/138 (0%)

#### Doctoral training program

##### (1) Curriculum

Research methodology, including quantitative and qualitative research methods, were the most common compulsory and elective subjects. Other commonly offered subjects (compulsory/ elective) were theory-related subjects, philosophy-related subjects, statistics, foreign language (English/Mandarin), attending research seminars, ethics, academic presentations and/or writing skills. Most of the students (75.5%) were satisfied with their curriculum and 74.8% felt satisfied with the quality of teaching in their programs.

##### (2) Academic presentation skills

Regarding presentation skills, 67.1% of the students indicated that there were some training components on academic presentation skills. The training was provided via seminars/workshops or by including it as a compulsory or elective subject. Almost all the students (98%) rated that training on academic presentation skills was important, but only 70.8% of the students were satisfied with their current training and only 66.7% felt confident with their academic presentation performance after receiving such training.

##### (3) Academic writing skills

Regarding academic writing skills, 66.4% of the students received trainings on enhancing their academic writing skills, and the most commonly used training approaches were seminars/workshops and compulsory/elective subjects. Training on academic writing skills was regarded as a very important training component by 95.8% of the students. Among those students who had received academic writing skills training, 71.9% were satisfied with it, although only 63.5% felt more confident about their academic writing skills after receiving such training.

##### (4) Teaching training experience/teacher education

Regarding teaching training, 55.9% of the students reported that no teaching training components were included in their doctoral program, as learning to teach was important for the students, particularly those who were planning to work as faculty members in a university setting. According to the other 44.1% of students who received teaching training, the types of teaching trainings were as follows: 1) mentoring/tutoring undergraduate nursing students (14.4%); 2) coordinating class discussions/group work of undergraduate or postgraduate nursing students (13.9%); 3) classroom lectures in undergraduate or postgraduate nursing courses (11.9%); 4) grading assignments/course papers/term papers for undergraduate or postgraduate nursing student courses (9.8%); 5) being examination supervisors for undergraduate or postgraduate nursing student courses (8.3%); 6) coordinating clinical placement of undergraduate or postgraduate nursing students (5.67%); and 7) mentoring/tutoring new doctoral students (5.2%). Among students who received teaching training, 93.8% believed that “teaching training in a doctoral program is important” but only 72.3% of them felt satisfied with the teaching training they received.

##### (5) Mobility opportunities

Less than half of the participants (45.8%) reported that mobility opportunities were provided by their institutions. Short-term student exchange programs, joint doctorate programs and internship programs were the most commonly provided mobility opportunities. Among students whose institution offered mobility opportunities, 89.9% agreed/strongly agreed that “mobility training opportunities are important for doctoral training”, but only 66.7% felt satisfied with the mobility opportunities provided by their institutions.

##### (6) Overall rating of the doctoral training components

Overall, eight out of ten students agreed that their critical thinking skills were enhanced after the doctoral training. However, only six out of ten (58.9%) and seven out of ten (67.4%) felt more confident in terms of writing research proposals and managing research projects respectively. The overall satisfaction of their doctoral training programs was high at 76.6% (Table 4).

#### Intellectual and cultural climate

The overall satisfaction with the intellectual and cultural climate was 76.1 and 68.1%, respectively. Less than 70% of the students agreed that their institutions were culturally diversified and had sufficient cultural interactions between staff and students (Table 4).

#### Facilities

Overall, 66.7% of the doctoral students felt satisfied with the facilities provided by their universities and nursing institutions (Table 4).

#### Overall study experiences and satisfaction

Through open-ended questions, it was identified that the biggest gain for the majority of the students was “having more research knowledge and skills” (*n* = 23, highest number of responses) followed by “learning to think critically” (*n* = 21 s highest number of responses). For the “biggest challenge during your doctoral study”, the most common response of the students indicated that “writing papers for publication” was the biggest challenge. Other commonly reported challenges included “thesis writing”, “language barriers, especially for writing and speaking”, “time management”, and “designing and implementing their research project”.

It was interesting to observe that students mentioned their relationship with the supervisor both as a major gain during their doctoral studies and as a major challenge at times. When the students were asked “what improvements in terms of the facilities in their institution they would like to see?”, the two most frequent expectations were improving library facilities and support, including more electronic databases, updated textbooks, better internet services, providing working stations and improving office facilities in working stations (printer, computer, etc.). Other frequently mentioned expectations were *soft facilities* such as more international and peer interaction programs, more financial support for research, more writing and presentation skills training and more technical support such as statistical support. For the overall satisfaction of their doctoral study, a three-part question was used (“If you had the chance to start your doctoral program again, would you select ‘the same university’, ‘the same field’ and ‘the same supervisor(s)’”. The results showed that 68.4, 85.7, and 78.9% of the doctoral students respectively would have retained their initial choices.

## Discussion

Two major models of nursing doctoral education have evolved over the years and have influenced the patterns of doctoral education worldwide. One model is prevalent in North America [21] and involves significant coursework and a research project while the other one is prevalent within the United Kingdom and Europe and is based mostly or entirely on a research thesis [22]. The study findings revealed that the doctoral nursing program model in most of the Asian countries is very similar to that of the American model, which values the importance of coursework alongside a significant research project. Although most of the participating institutions in ESEA countries had domestic and/or international collaborations such as the student exchange programs, the students still rated that there was room for improvement on the mobility opportunities. The collaborative arrangements between academics and doctoral students allow partners to combine respective strengths in achieving the goals and the numbers of qualified faculty [23]. More effective collaborations and exchange programs should be established and performed to increase the relevance of doctoral nursing education to changing global healthcare concerns [1].

While more than half of the students reported that they received training on academic presentation skills and writing skills during their doctoral studies, satisfaction with the training was not as high as in other areas surveyed, and around one third of the students still did not feel confident with their academic presentation and writing skills. In addition to coursework or workshops and seminars, other approaches including more opportunities for attending local/national/international conference presentations or writing groups (e.g., student-run manuscript club) could be adopted. More importantly, the effectiveness of such training should also be evaluated.

The programs surveyed were primarily traditional research-based PhD programs, although a small number of professional doctorate programs, similar to Doctor of Nursing Practice in the United States (DNPs), started appearing in three countries [24]. A shift in the development of professional programs was observed, with several of the institutions in the study considering to set up such a program; this is a trend that is expected to continue. The expansion of such programs may compete for student numbers with PhD programs; as their orientation should be different, this may create in the future role confusion and negatively impact academic leadership [24]. Indeed the professional doctorate programs assessed differed very little from the PhD programs in this survey as the coursework was similar to that of the PhD programs, there was no strong clinical component and a research project of the same extent with the PhD one was expected to be carried out. Finally, no university in the surveyed countries offered an option of PhD by publications, a recent trend in some western countries.

High supervision workload was identified in the ESEA countries, with one supervisor having five or more full-time doctoral students at the same time, when all supervisors are also engaged in full teaching duties. The possible reasons for this might be faculty shortages and an aging faculty. However, it is suggested that each supervisor should have no more than three doctoral students as having more than three doctoral students can reduce the capacity of the supervisor in supervising and time spend on doing research [25] although this number is debatable. Having less time for doctoral students can adversely affect the relationship between the supervisor and student, since this relationship is a very important factor in student decisions to continue or withdraw from their doctorate [26]. Nevertheless, the majority of the students hold positive experiences of the research supervision provided by their supervisors and they generally felt satisfied with the supervision quality. The findings are consistent with the study conducted by Nagata [6], where the doctoral students in Japan generally hold very positive attitudes towards the quality of the academic staff for doctoral supervision, which partially reflects “Asian culture that honors and respects teachers” (p. 366).

In addition, more than half of academic members in the nursing schools surveyed were from other health science disciplines as well as from non-health disciplines such as data science and informatics science. Non-nursing faculty members could promote interdisciplinary collaboration. However, including a non-nursing academic member to be the supervisor of doctoral nursing students might increase research projects that have less relevance and impact on the nursing field alongside compromising research in nursing sciences, with the new scholars not being socialized in nursing [27]. It raises also a concern regarding the preparation of non-nurse supervisors when they are assigned to be supervisors [28]. Besides, nursing academic staff and doctoral students, particularly for universities in China, South Korea and Japan, are mostly domestic staff and students, and this may have decreased the students’ satisfaction with cultural diversity and interactions. Cultural diversity therefore should be considered when recruiting academic members and students. Some countries are using a scholarship system to attract international students or waive fees for international students in combination with country agreements, such as in Hong Kong or Thailand. These are appropriate ways, when resources allow it, to enhance the cultural diversity of doctoral students.

The doctoral nursing programs in ESEA countries, while they emphasized more on research (expectedly as they are research-based degrees), they placed less emphasis on other areas of knowledge such as teaching preparation. Insufficient teaching preparation might cause difficulties for the graduates’ career pathways because many nursing doctoral graduates pursued their future career as an academic working in higher education institutions and teaching would be one of the key duties. Teaching training therefore can be an important component in doctoral nursing training programs [29]. The changing purpose of a doctoral degree has been debated widely and authors argue that the predominant focus of a doctoral degree on research skills need to be complemented with a clear focus on purpose of and intentions from a doctoral program [30, 31]. It is clear that there is a discordance between the requirement of a PhD degree for an academic faculty position and limited formal preparation for this role in a traditionally research-based degree, despite recommendations from organisations and studies for pedagogical preparation of nurses during their doctoral studies [31]. The question of the purpose of a PhD degree should be considered more carefully in the future in terms of how doctoral candidates are prepared for their future careers.

Although the students hold generally positive study experiences, they encountered some challenges during their doctoral study journey, with language barriers mentioned in open comments as a significant challenge, as students needed to publish their work in English language international journals or present their work in international conferences. For some Asian universities in China, Taiwan, Japan and South Korea, the language of instruction for most of the doctoral program-related subjects is their home language with only few subjects using English. However, doctoral students from those countries, particularly China and Taiwan, are required to have some English publications as compulsory requirements for attaining their doctoral degree. These responses also revealed that there is a strong need to enhance the English proficiency of doctoral nursing students in Asian countries. In addition to further enhancing the existing English courses, more international exchange opportunities with universities in other countries, and the development of new doctoral nursing subjects/courses using English as the main language of instruction could be options to help students overcome language barriers, particularly in this era of globalization and international movement of health professionals. In addition, for most of the institutions, the improvement in administrative support and research-related facilities failed to meet the needs of the fast expansion of the intake of doctoral nursing students in recent years. Improvements of the research facilities would be necessary to better support learning and research activities of doctoral nursing students. Furthermore, delaying in completion and inadequate financial support from schools were two other challenges. This finding was in line with previous literature, which found doctoral nursing students usually complete programs later than students in other disciplines [31, 32]. Extensions in the study period not only create more stress but also a financial burden to students. This issue not only indicates a need for further investigation on this topic but also that institutions should develop better financial support mechanisms for their students.

This study has several limitations. Firstly, due to the nature of a cross-sectional survey, we could not determine factors that affect doctorate completion in postgraduate students in the target population. A longitudinal study or a qualitative study could elaborated more on this question. Secondly, a large proportion of respondents were from Japan (although this is in accordance with the large number of PhD programs available in Japan), which might limit the generalizability of the findings, and under-represent some countries. Also, self-reported data may introduce personal biases and hence more objective data in a future study may enhance the generalizability of self-reported findings. Biases in a survey as well as incomplete data in some later sections of a survey can be minimized when survey items are randomized each time or alternated, and this was not done in the present e-survey. Furthermore, the completion rate in the open ended questions was relatively low. While this is not unusual in surveys, as respondents require more commitment to write a free-form response, it may introduce biases in the data. Finally, an interesting question would have been the differences in content and processes between PhD and professional doctorate programs, but the small number of the latter participants prevented us from making a more in-depth analysis on this important issue.

## Conclusion

The study reports on 196 doctoral nursing students in 11 South- and South East Asian countries. This survey is the first study to explore the current characteristics of nursing doctoral education programs and students’ study experiences of and satisfaction with those programs in ESEA countries and regions. Our descriptive study provides information that might be useful to compare in the future with similar programs in other regions or continents. Considering the limited resources in some countries, the preparation of supervisors, the challenges experienced by students and the overall quality of nursing doctoral programs in the region, there is a need for developing and setting standards for quality doctoral education., As the issues reported in this study may be similar to those of other such programs outside this region, minimal quality criteria and educational standards could be agreed upon internationally. Societies such as the East Asian Forum of Nursing Scholars (EAFONS, http://eafons.org/index.html) and the International Network for Doctoral Education in Nursing (INDEN, http://indenglobal.org/) can play a major role in enhancing quality of doctoral programmes internationally by streamlining requirements and standards. An accreditation and/or recognition system of institutes offering nursing doctoral programs may be one such option. These societies should also work proactively to develop guidelines to maintain or enhance quality indicators that are relevant to each country and also identify strategies for influencing policies relevant to doctoral education.

## Data Availability

Data and materials of the study are available to others upon request.

## Abbreviations

DNP: Doctor of Nursing Practice
EAFONS: East Asian Forum of Nursing Scholars
ESEA: East and South East Asia
INDEN: International Network for Doctoral Education in Nursing
PhD: Doctor of Philosophy
